# Microfluidic Formation of Monodisperse Coacervate Organelles in Liposomes

**DOI:** 10.1002/anie.201703145

**Published:** 2017-07-18

**Authors:** Nan‐Nan Deng, Wilhelm T. S. Huck

**Affiliations:** ^1^ Radboud University Institute for Molecules and Materials Heyendaalseweg 135 6525 AJ Nijmegen The Netherlands

**Keywords:** artificial organelles, coacervates, liposomes, microfluidics, phase transitions

## Abstract

Coacervates have been widely studied as model compartments in protocell research. Complex coacervates composed of disordered proteins and RNA have also been shown to play an important role in cellular processes. Herein, we report on a microfluidic strategy for constructing monodisperse coacervate droplets encapsulated within uniform unilamellar liposomes. These structures represent a bottom‐up approach to hierarchically structured protocells, as demonstrated by storage and release of DNA from the encapsulated coacervates as well as localized transcription.

Complex coacervation is the associative phase separation of oppositely charged polyelectrolytes.^1^ This form of liquid–liquid phase separation is a powerful means of compartmentalization and has been explored extensively as a protocell model for the construction of artificial cells or organelles.^2^ Protocells based on simple and complex coacervates have been shown to display interesting properties, including enhanced enzyme catalysis,^3^ selective partitioning of biomolecules,^4^ and model crowded environments.3e, 5 Importantly, coacervates can also be found in living cells, for example P granules,^6^ stress granules,^7^ and Cajal bodies.^8^ Increasing evidence suggests that these compartments originate via liquid–liquid phase separation (LLPS) of (intrinsically disordered) proteins and RNA^9^ and play an important role in cell structure and functions involving RNA metabolism.^9^ The prevalence and importance of coacervates in biology, in combination with their relevance as artificial compartments, inspired us to explore the formation of well‐defined functional coacervates encapsulated within membranous structures.

Recently, microfluidic approaches to multicompartment^10^ and core–shell vesicle structures^11^ have been reported as advanced artificial cell models.10a, 11a Herein, we demonstrate the embedding of coacervate droplets into liposomes. These internal organelle‐like compartments allow for a high level of control over the spatial organization of biochemical processes. Similar liposomes containing a synthetic polymer‐based aqueous two phase system (ATPS), that is, polyethylene glycol and dextran (PEG‐DEX),^12^ have already been reported through a bulk hydration of dried lipid membranes and shown diverse cell‐like properties, such as microcompartmentalization,12a,12b protein relocalization in response to stimuli,12c and asymmetric vesicle division.12d However, more bio‐relevant coacervate systems have not been achieved in phospholipid vesicles until now, probably because complex coacervates are highly charged, which interferes with conventional liposome preparation methods.^13^ Additionally, typical liposome formation methods lead to polydisperse structures, give low yields, and show inefficient encapsulation. Furthermore, ATPS phase transitions often require undesired heating and cooling steps (the temperature applied is as high as 50 °C)12a or osmotic shocks,^14^ which are incompatible with biological processes and cause instability of liposomes.

In this paper, we report a robust and straightforward microfluidic strategy for encapsulating the coacervate systems into liposomes to create artificial non‐membrane‐bound sub‐compartments. This method shows exceptional robustness, flexibility and controllability, and results in monodisperse structures. To show the potential as artificial organelles/nucleoids, we performed the thermal‐responsive reversible coacervation of macro‐ions in liposomes to collect and release DNA molecules, and the spatial organization of in vitro transcription (IVTx). This work represents a bottom‐up approach to building model artificial/protocells with high‐order internal architectures.

Prior to microfluidic encapsulation, we tested the complex coacervation of a range of polycations, such as poly‐l‐lysine (pLys) and poly‐l‐arginine (pArg), and polyanions, such as RNA and ATP, in bulk, (Figure S1, see the Supporting Information for full experimental details). As expected, all of these systems can form complex coacervates but result in polydisperse structures (Supporting Information, Figure S2). To show they are indeed coacervates rather than precipitates, we centrifuged the sample and obtained two clear phases (Supporting Information, Figure S3). The bottom phase consists of the fused coacervate droplets, as they have a higher density. Coacervate droplets move and fuse with each other under an interfacial tension gradient created by water evaporation, which is a clear sign that they are liquid‐like (Supporting Information, Figure S4). To investigate diffusion inside the coacervates, we used fluorescence recovery after photobleaching (FRAP). Upon photobleaching of a section of coacervate droplets, the fluorescence recovered rapidly (Supporting Information, Figure S5), demonstrating that coacervate droplets are liquid.

To prepare monodisperse coacervate droplets in monodisperse liposomes, we employed a microcapillary based microfluidic device^15^ with core–shell inlets to load polycations and polyanions (W1 and W1′) into double emulsion droplets. As Figure 1 a shows, when two oppositely charged polyelectrolytes were simultaneously injected into the device, a large number of (sub)micron coacervate droplets formed (Figure 1 a, b1), which subsequently became encapsulated into W/O/W double emulsion droplets (Figure 1 b1, Movie S1). The coacervate system that we first tested was pLys (W1, 10 mm) and ATP (W1′, 10 mm). In a typical experiment, we used a mixture of chloroform and hexane (30:70, v/v) containing 5.0 mg mL^−1^ l‐α‐ phosphatidylcholine (egg PC) as the middle oil phase (O) and an aqueous solution of 10.0 wt% PVA and 0.3 wt% F‐68 as the outer water phase (W2, see the Supporting Information for full details).10a As the solvents evaporated, the as‐prepared double emulsion templates underwent a dewetting transition, generating uniform unilamellar liposomes (Figure 1 b2 and Supporting Information, Figure S6).10a Meanwhile, the encapsulated coacervate droplets coalesced into a single large droplet within approximately 15 min (Figure 1 c, Movie S2).

**Figure 1:**
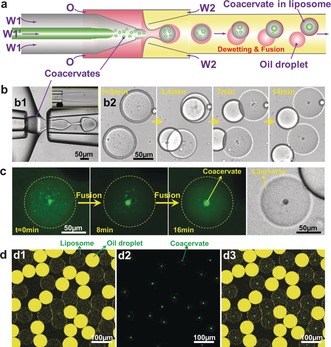
Encapsulation of coacervates into liposomes. a) Illustration and b) images of the microfluidic preparation of W/O/W double emulsions with coacervates as well as relevant dewetting transition and fusion process to form a liposome with a coacervate droplet. Inset in (b) shows the mixing of W1 and W1′ in microchannels. c,d) Confocal images of the fusion of small coacervates into a big coacervate in liposome (c) and as‐prepared uniform liposomes containing monodisperse coacervate droplets (d, panel d1 shows liposomes and residual oil droplets with excess lipids; panel d2 shows the labelled coacervates; panel d3 is the overlay of d1 and d2). Polycation=poly‐l‐lysine, polyanion=ATP.

Interestingly, we found that all the coacervates are located precisely in the middle of the liposomes (Figure 1 d3 and the Supporting Information, Figure S6). This can be explained by considering that the density of the coacervates is higher than that of surrounding solution (Figure S3), so the coacervate droplets sink down to the bottom of spherical liposomes (Supporting Information, Figure S7). Observed from the top, all the coacervates are in the middle of liposomes, while observed from the side, they locate at the bottom of liposomes (Supporting Information, Figure S8).

Our method is flexible and highly controlled and produces monodisperse structures in high throughput (as high as 10^3^ s^−1^) and high yields (more than 90 % of double emulsion droplets form liposomes).10a As Figure 1 d shows, both liposomes and inner coacervate droplets show high monodispersity; their mean diameters are, respectively, 78 μm and 10 μm and the coefficients of variation are 4 % and 11 %, respectively (Supporting Information, Figure S9). To show the flexibility and controllability of our method, we successfully achieved a number of coacervate systems in liposomes (Supporting Information, Table S1), including polyuridylic acid (polyU)/spermine, ATP/pLys, and coenzyme A (CoA)/pArg (see the Supporting Information for full details), as well as a synthetic ATPS, PEG‐DEX (Supporting Information, Figure S10). We note that in some systems W1/W1′ jetting can be directly sheared into single water‐in‐water droplets and encapsulated into double emulsion droplets (Figure S10). Moreover, the sizes of inner coacervate droplets and outer liposomes can be easily tuned by changing the applied flow rates (Supporting Information, Figure S11) and the concentrations of polyelectrolytes (Supporting Information, Figure S12). By changing the surfactant concentration in the outer phase, the dewetting time can be adjusted as well (Supporting Information, Figure S13). Importantly, this method does not require heating and cooling steps or osmotic shocks, making it compatible with biological research.

Non‐membrane‐bound organelles have been recognized as an effective compartmentalization strategy for the cell as they facilitate localized high concentrations of specific proteins and substrates. Furthermore, these structures are inherently dynamic and can form/disappear in response to changes in the cellular environment. To demonstrate that we can mimic these phase transitions, we conducted the dynamic dissolution and re‐assembly of coacervates in liposomes (Figure 2 a). As reported previously, complex coacervation can be well tuned through charge ratios,^16^ pH,2c temperature,^17^ and light.^18^ Herein, we show the dynamics by using complex coacervates composed of low‐complexity RNAs and short polyamines, which show reversible coacervation in response to temperature changes (higher or lower than the lower critical solution temperature (LCST)).^17^ We encapsulated polyU and spermine coacervates (LCST≈20 °C)^17^ into liposomes and observed them as the temperature changes (Figure 2 a). As Figure 2 b,c shows, when the temperature was lower than the LCST, the coacervates gradually dissolved. But when the temperature was increased above the LCST, a myriad of small coacervates emerged again and finally fused into a large coacervate over time (Supporting Information, Figure S14 and S15, and Movie S3). The striking temperature‐dependent behavior is fully reversible, could be repeated over five rounds, and provides an elegant starting point for studying dynamic biological compartmentalization.

**Figure 2:**
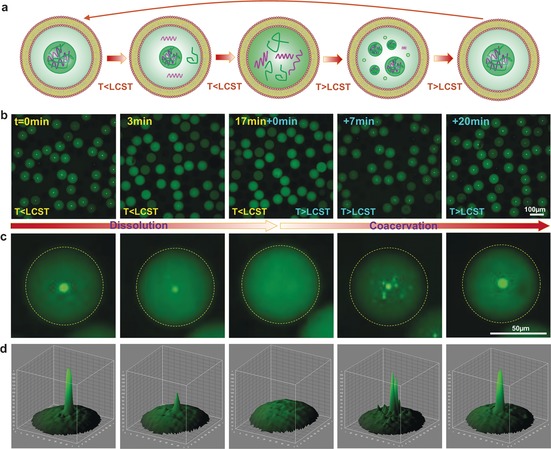
Thermal dynamics of the membraneless organelle‐like compartment in liposomes. a) Illustration and b) confocal images of dissolution and coacervation of the coacervate droplets inside liposomes over time as temperature changes. c) Magnified views of a single liposome with dynamic artificial organelles and d) the relevant 3D fluorescence intensity profiles. Polycation=spermine, polyanion=polyU RNA.

Next, we encapsulated labelled double‐stranded DNA molecules into liposomes together with polyU/spermine complex coacervates (Figure 3 a). As Figure 3 b3 shows, DNA molecules partition efficiently into the artificial organelles because of electrostatic interactions and a low dielectric constant.2c, 4, 16b DNA most likely competes with polyU for interactions with spermine in the coacervates, probably displacing some of the polyU during partitioning. However, the DNA is at very low concentration (5 nm) and this partitioning does not have any consequences for coacervate formation or stability. It should be noted that DNA alone cannot form coacervates at these low concentrations. The DNA localization follows the dynamics of coacervation and dissolution—showing a storage and release function in the liposomes (Figure 3 b–c).

**Figure 3:**
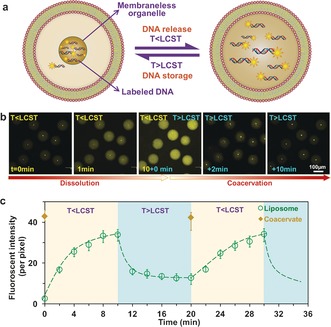
Release and storage of labelled DNA molecules in artificial organelles. a) Illustration and b) confocal images show thermally triggered release and storage of labelled DNA molecules in the coacervates within liposomes. c) Kinetics of localized fluorescence of labelled DNA in liposomes and coacervate droplets when temperature was switched under or above coacervate LCST. Polycation=spermine, polyanion=polyU RNA.

The compartmentalization of biomolecules allows us to build spatially functional artificial/protocells. To prove this concept, we achieved in vitro transcription (IVTx) inside the non‐membrane‐bound sub‐compartments. As Figure 4 a,b shows, the complex coacervates composed of spermidine and polyU together with IVTx components (see the Supporting Information, IVTx in artificial nucleoids, for details) were encapsulated into liposomes to prepare artificial cells with a nucleoid‐like structure. To visualize the RNA generation, we coded the DNA templates with a sequence for Spinach2 aptamer that can bind 5‐difluoro‐4‐hydroxybenzylidene imidazolinone (DFHBI) to form a fluorescent complex of Spinach2–DFHBI (Figure 4 a, last image);^19^ both DFHBI and Spinach2 are non‐fluorescent until binding occurs. The sequence of confocal images in Figure 4 c–e shows high fluorescence in the coacervates due to RNA synthesis, while a lower signal was observed in the surrounding water shells. To confirm that the IVTx does not occur outside the coacervate droplets, we removed coacervates from mixtures of coacervates and IVTx mix by centrifugation, and then recorded the reaction in supernatants using a plate reader. The results show that no increase of fluorescence was observed (Supporting Information, Figure S16). As the DNA partitions into the coacervates and there is no clear fluorescence increase outside the coacervates, it is clear that transcription exclusively occurs in the coacervates.

**Figure 4:**
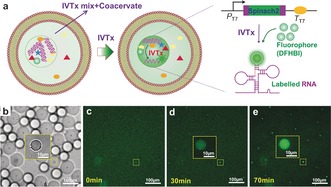
Spatial organization of bio‐reaction in artificial organelles. a) Illustrations of IVTx in coacervate droplet in liposome and the working principle of detection of generated RNA using aptamer Spinach2 and dye DFHBI. b) Optical image of as‐formed liposomes containing coacervate droplet (Polycation=spermidine, polyanion=polyU RNA) and IVTx mix. c–e) Confocal images show RNA generation in coacervates over time.

In summary, we have presented a microfluidic strategy for fabricating monodisperse coacervate compartments in monodisperse liposomes. The control of complex enzyme‐catalyzed reactions in cytomimetic compartments represents a key step towards artificial cells. The cytomimetic structures allow us to mimic diverse intracellular activities, such as thermally responsive reversible compartmentalization, controlled storage and release of genetic molecules, and spatial organization of bioreactions. Importantly, this work represents a bottom‐up approach to building artificial/protocells with high‐order compartmentalization, which we hope will facilitate research on cell‐mimicking and artificial living systems, and provide novel insights into the mechanisms of how protocells spatiotemporally control chemical reactions.

## Conflict of interest

The authors declare no conflict of interest.

## Supporting information

As a service to our authors and readers, this journal provides supporting information supplied by the authors. Such materials are peer reviewed and may be re‐organized for online delivery, but are not copy‐edited or typeset. Technical support issues arising from supporting information (other than missing files) should be addressed to the authors.

Supplementary

Supplementary

Supplementary

Supplementary
