# Supplementary material for: Microfluidic Formation of Monodisperse Coacervate Organelles in Liposomes
Source: Angew Chem Int Ed Engl. 2017 Jul 18;56(33):9736–40. doi: 10.1002/anie.201703145 (PMC5601218; doi:10.1002/anie.201703145)
Supplement: Supplementary file 1 — Supplementary [file ANIE-56-9736-s001.pdf]

Angewandte Chemie
Eine Zeitschrift der Gesellschaft Deutscher Chemiker

Supporting Information

Microfluidic Formation of Monodisperse Coacervate Organelles in Liposomes

Nan-Nan Deng and Wilhelm T. S. Huck**

anie_201703145_sm_miscellaneous_information.pdf

Supporting Information

Table of Contents

Part I. Supplementary Experimental Section 2

1. Materials 2

2. Coacervation in bulk 3

3. Microfluidics 4

3.1 Microfluidic devices 4

3.2 Manipulation 4

4. Reversible compartmentalization inside liposomes & DNA storage and release in dynamic artificial organelles 5

5. IVTx in artificial nucleoids 6

Part II. Supplementary Figures S1-S16 and Table S1 8

Part III. Supplementary Movies S1-S3 20

Part IV. Supplementary References 21

WILEY-VCH

Part I. Supplementary Experimental Section

1. Materials

To prepare monodisperse coacervates, a number of polycations and polyanions were used (Figures S1), including polyuridylic acid (polyU)/spermine, polyU/spermidine, adenosine triphosphate (ATP)/poly-L-lysine (pLys), ATP/poly(diallyldimethylammonium chloride) (PDDA), and coenzyme A (CoA)/poly-L-arginine (pArg). All the chemicals were purchased from Sigma-Aldrich. To prepare monodisperse liposomes with coacervate droplets from templates of W/O/W double emulsions, two aqueous solution respectively conatining polycations and polyanions were used as two inner water phases (W1 and W1'); a mixture of chloroform and hexane (30:70, v/v) containing 5 mg mL^{-1} L-α-phosphatidylcholine (egg PC, Avanti Polar Lipids) was used as middle oil phase (O); an aqueous solution with 10 wt.% polyvinyl alcohol (PVA, M_W= 13,000-23,000 g mol^{-1}, 87-88% hydrolysed, Sigma-Aldrich) and 0.3-1.0 wt.% Pluronic® F-68 (ThermoFisher Scientific) was utilized as outer water phase (W2). To visualize as-formed coacervates and liposomes, the coacervates (green drops in FIgures 1 and Figure 2) were labelled by fluorescein isothiocyanate labelled lysine-serine polycations (FITC-pLys, Mw 2367 g mol^{-1}) (Ref. 16a in the main text); lipid bilyer membranes were labelled by 1,2-dimyristoyl-sn-glycero-3-phosphoethanolamine-N-(lissamine rhodamine B sulfonyl) (ammonium salt) (Rh-PE). To prepare PEG/DEX ATPS droplets, 5, 10, 15 wt.% polyethylene glycol (PEG, M_W= 6,000 g mol^{-1}, VWR) and 10, 15 wt.% dextran (DEX, Mw. 500000 g mol^{-1}, Sigma-Aldrich) were used. The ATPS droplets were labelled by fluorescein isothiocyanate-dextran (FITC-Dextran, M_W= 40,000 g mol^{-1}, Sigma-Aldrich).

WILEY-VCH

2. Coacervation in bulk

Prior to microfluidic encapsulation, we first tested the complex coacervation with diverse polycations and polyanions in bulk (Figures S1) including 0.05 wt.% polyU and 0.5 wt.% spermine (in 5 mM HEPES pH 7.6 and 1 mM Mg^{2+}) (Ref. 17 in the main text), 10 mM ATP and 10 mM pLys, 5 mM ATP/20 mM PDDA at pH 8.0, and 10 mM CoA and 10 mM pArg. As expected, all of these systems can form complex coacervates, but result in polydisperse and unstable structures (Figure S2). To prove there is indeed a liquid-liquid phase separation and the fluorescent spots we observed are coacervates rather than precipitates, we did three types of experiments (Ref. 1 and 17): 1) centrifuging; 2) interfacial tension-directed movement and fusion; 3) fluorescence recovery after photobleaching (FRAP) experiment. Firstly, 50 µL mixture containing 50 mM ATP, 50 mM pLys and 5 mg mL^{-1} FITC-pLys was prepared in an eppendorf tube (0.2 mL), then centrifuged at 14 000 rpm for 2 min (MiniSpin Plus, Eppendorf). To further confirm the fluidity of the coacervates, we preformed interfacial tension-directed movement and fusion. A drop of coacervate solution was placed between a glass slide and a cover glass. The movement and fusion of coacervate droplets were recorded by a spinning disk confocal laser microscopy (iXon3, Andor) in both fluorescence and bright fields. To investigate diffusion inside the coacervates, FRAP experiments were carried out on an Olympus IX81 confocal microscope, equipped with an Andor iXon3 camera, Andor 400-series solid-state lasers, a Yokogawa CSU-X1 spinning disk, and an Andor FRAPPA photobleach module. Recovery of the fluorescence intensity was recorded every second.

WILEY-VCH

3. Microfluidics

3.1 Microfluidic devices

The microfluidic devices used here are assembled from round and square glass capillaries reported by the Weitz group (Ref. 15 in the main text). To prepare liposomes with coacervate droplets from the templates of double emulsion droplets, we upgraded the devices with two-inlets for pumping two water phases of polycations and polyanions at the same time. Briefly, a cylindrical microcapillaries of outer diameter 170 µm, inner diameter 100 µm were inserted into a bigger cylindrical microcapillary of outer diameter 960 µm, inner diameter 400 µm, which was precisely tapered to achieve orifice sizes of about 40-60 µm in diameter by using a capillary puller (PN-31, Narishige) and a microforge (MF-830, Narishige) and used as inlet in device assembly. Another same cylindrical microcapillary were precisely tapered to achieve orifice sizes of about 80-120 µm in diameter as outlet. The microcapillary with smaller tip modified by trimethylsilyl chloride (Sigma-Aldrich) into hydrophobic was used for flowing inner phase, while the capillary with larger diameter was treated by 2-[methoxy (polyethyleneoxy) propyl] trimethoxy silane (Gelest, Inc.) to render its surface hydrophilic, and used as the collection tube. Both of two cylindrical capillaries were inserted into a square capillary of inner diameter 1.00 mm from its two opposite ends. The gaps between the square capillary and round capillary are used as two channels for flowing middle and outer phase as shown in Figure 1a. Lastly, dispensing needles used as inlets of fluids were connected at the junctions between capillaries or their ends by using a transparent 5 minute® Epoxy (Devcon).

3.2 Manipulation

To generate the double emulsions, all fluids were pumped into the capillary microfluidic devices by using syringe pumps (PHD 2000 series, Harvard Apparatus) at desired flow rates. Typical flow rates of the two inner phases, middle and outer phases are 150, 150, 500, and 5000 µL h^{-1}, respectively. The formation process of emulsion drops was monitored by using an

inverted optical microscope (IX71, Olympus) equipped with a high-speed camera (Miroex4, Phantom, Vision Research). The freshly prepared emulsion templates were collected in a semi-enclosed silicone isolation chamber (diameter 9 mm, height 0.12mm, SecureSeal^{TM}) covered with a glass coverslide for further characterization. The resultant labelled liposomes containing labelled coacervates were observed by a confocal laser scanning microscope (CLSM) (SP8x, Leica) or a spinning disk confocal laser microscope (iXon3, Andor).

4. Reversible compartmentalization inside liposomes & DNA storage and release in dynamic artificial organelles

To carry out the dissolution and coacervation of coacervates inside liposomes, polyU RNA and polyamine (spermine or spermidine) were used here to prepare thermal-dynamic coacervates according Ref. 17 in the main text, in which the final concentrations of polyU RNA and spermine/spermidine were respectively 0.05 wt.% and 0.5 wt.%. In some experiment, 5 mM HEPES (4-(2-hydroxyethyl)-1-piperazineethanesulfonic acid) and 1 mM Mg^{2+} were added. For visualize the dynamic process, we also added some Spinach2-DFHBI (expressed from IVTx) into the polyU RNA phase before mixing. To show the storage and release of DNA in artificial organelles, 5 nM labelled double-stranded DNA (prepared from mixing of ATP, T4 DNA ligase, oligo-A (5'-TACAAGACAC-3'), oligo-B (5'-GACGGGAAG-3'), oligo-C (5'-p-TACAAGACAC-3') and a molecular beacon (MB-2: 5'-CCTCTCCGT···GAGAGG-3')[1] was added into the coacervate system before microfluidic encapsulation. For the control of temperature in dissolution and coacervation experiments, we used a Temperature Controller (E5CN, Omron) to heating up the samples to 25 ^{o}C and a Liquid Cooling System (EX2-755, Koolance) to cool down the samples to 16 ^{o}C. Meanwhile, the temperature of samples was also monitored by a IR Thermometer (IRT-350, Basetech).

5. IVTx in artificial nucleoids

For the *in vitro* transcription (IVTx) reactions, we used a reaction buffer without any cell lysate for cell-free transcription reactions as previously reported.[2] The reaction buffer consisted of 50 mM 4-(2-hydroxyethyl)-1-piperazineethanesulfonic acid (HEPES, pH 8.0), 3 mM guanosine triphosphate, 1 mM each of adenosine triphosphate, cytidine triphosphate and uridine triphosphate, 0.66 mM spermidine, 0.5 mM cyclic adenosine monophosphate, 0.22 mM nicotinamide adenine dinucleotide, 0.17 mM coenzyme A, 20 mM 3-phosphoglyceric acid, 0.045 mM folinic acid, 0.13 mg ml^{-1} transfer ribonucleic acid and 1 mM of each amino acid. This reaction buffer was premixed and stored in aliquots at -80 ^{o}C after flash-freezing. To visualize and real-time detect the RNA generation, DNA template coding for Spinach2 aptamer with tRNA scaffold (15 nM) was used. To this reaction buffer we also supplemented 100 U of RNase inhibitor (human placental, NEB), 60 uM of 5-difluoro-4-hydroxybenzylidene imidazolinone (DFHBI) (Tocris), 70 mM potassium glutamate, 15 mM magnesium glutamate, 1.5 U of pyrophosphatase, inorganic (yeast, Sigma), 300 U of T7 polymerase.

To perform the IVTx in coacervates in liposomes, we used a mixture of IVTx mix and coacervate solution (100 µL 1.0 wt.% polyU, 200 µL 50 mM spermidine and 160 µL IVTx mix mentioned above) as the inner water phase to prepare liposomes. The microfluidic preparation process was carried out at about 18 ^{o}C. The collected samples were incubated at 28 ^{o}C and monitored for 2 hours by a spinning disk confocal laser microscopy (iXon3, Andor) equipped with a Temperature Controller (E5CN, Omron). The detection interval was 5 min, and the exposure time was 500 ms.

To confirm that the IVTx occurs inside the coacervate droplets, we first prepared mixtures of coacervates and IVTx solution as mentioned above at 15 ^{o}C, then increased the temperature to 25 ^{o}C to induce the coacervation. Subsequently we removed the complex coacervates by

WILEY-VCH

centrifuging at 10000 rpm for 5 min (MiniSpin Plus, Eppendorf), and finally recorded the reaction in the supernatants using a plate reader (Infinite M200 PRO, Tecan). The positive control (only IVTx mix) was carried out as well. The temperature in the plate reader was set at 30 ^{o}C.

WILEY-VCH

Part II. Supplementary Figures S1-S16 and Table S1

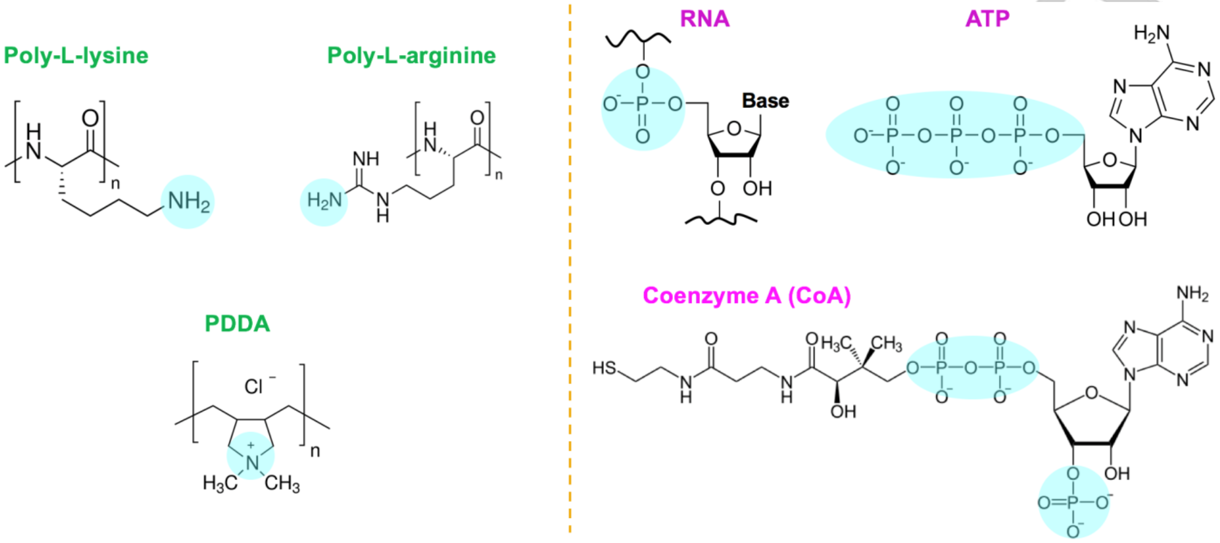

Figure S1. Examples of polycations and polyanions used in this paper to form complex coacervates.

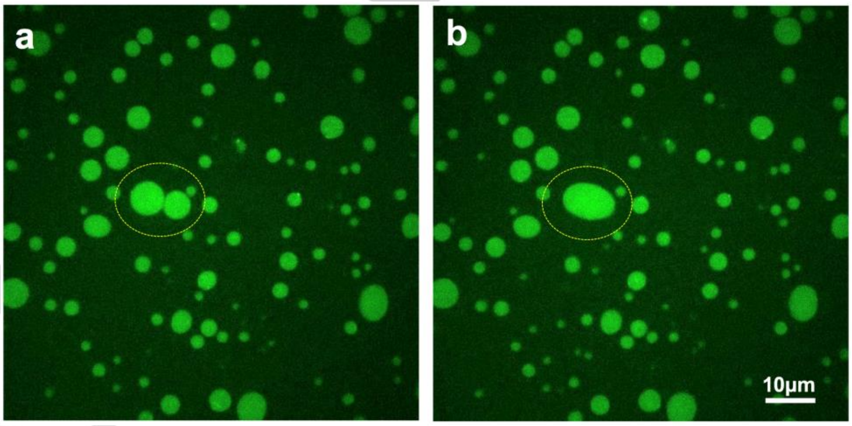

Figure S2. Confocal images of the coacervates prepared in bulk from ATP/pLys and a fusion process. Scale bar, 10µm.

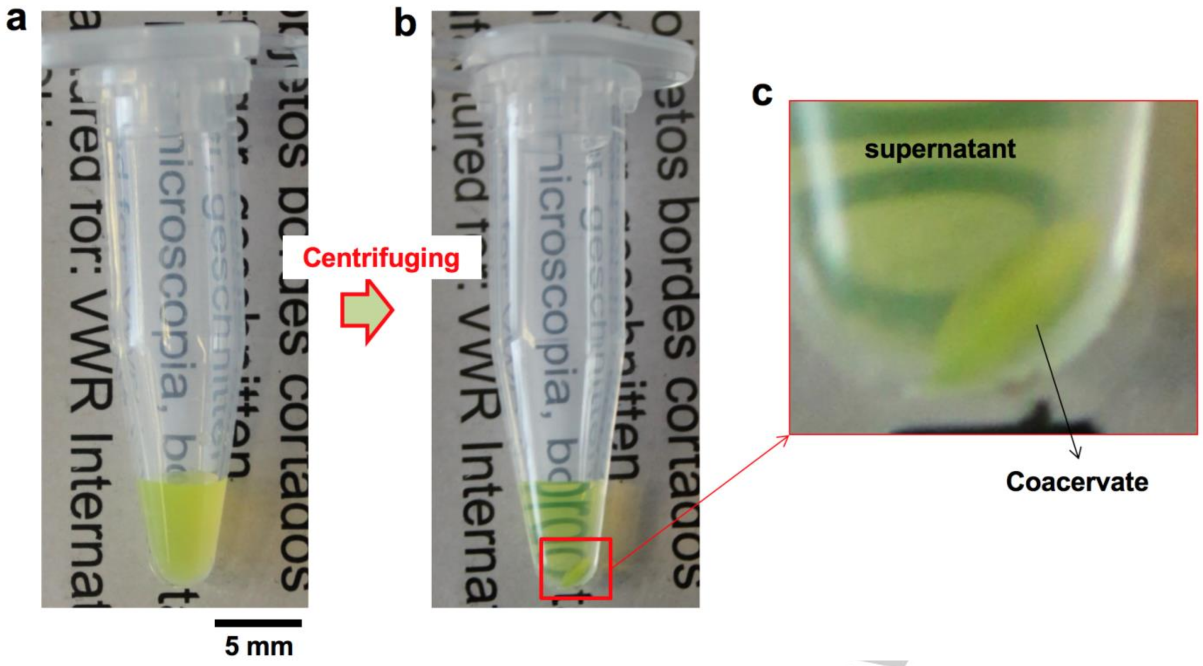

Figure S3. Optical photographs of the coacervate samples (50 mM ATP and 50 mM pLys) prepared before (a) and after (b) centrifuging and an enlarge image showing the merged coacervate (c). Scale bar, 5 mm.

WILEY-VCH

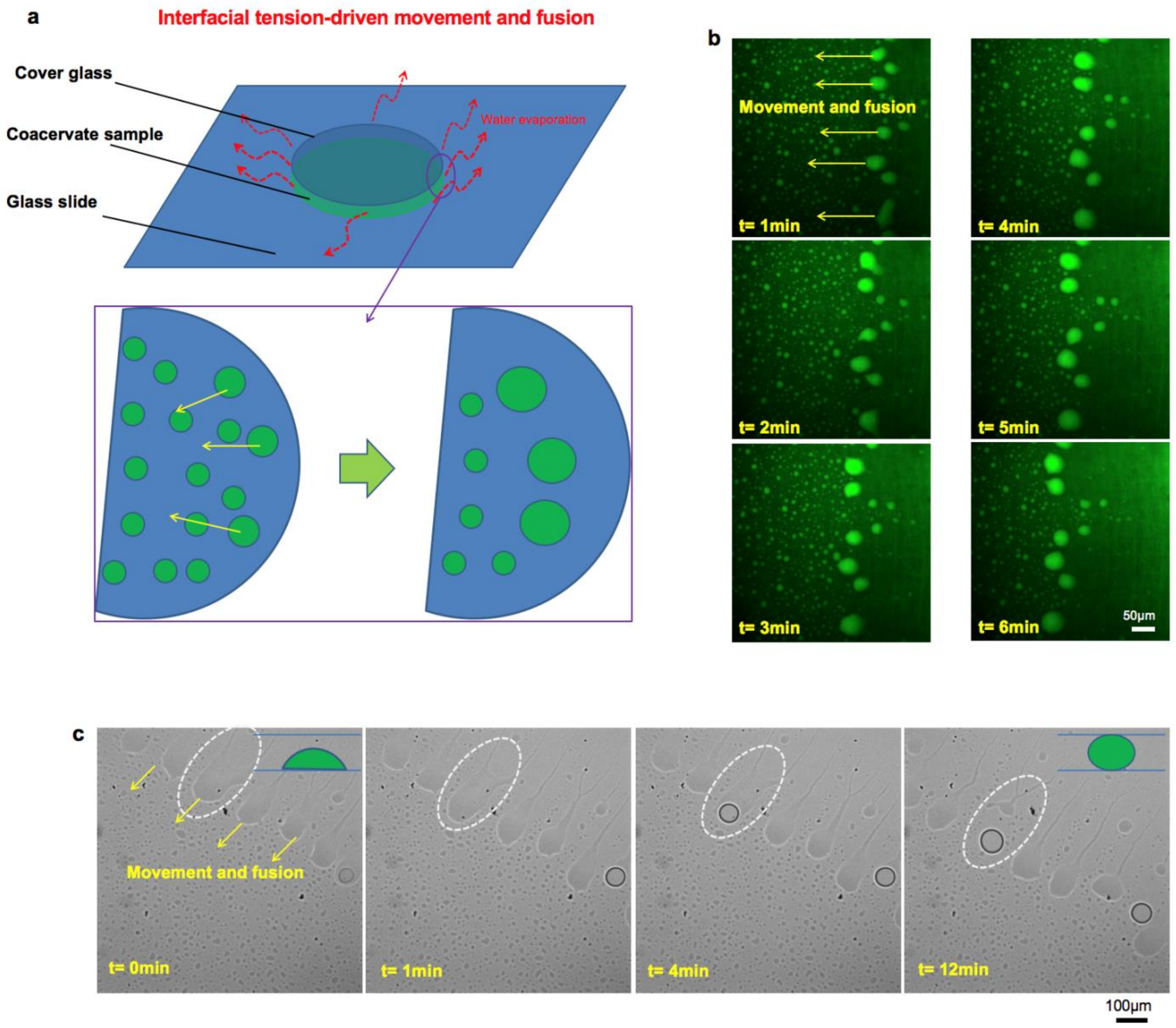

Figure S4. Schematics (a), confocal images (b) and optical photographs (c) of interfacial tension-directed movement and fusion of coacervate droplets. Coacervates consist of 10 mM ATP and 10 mM pLys as well as 0.2 mg mL^{-1} FITC-pLys.

WILEY

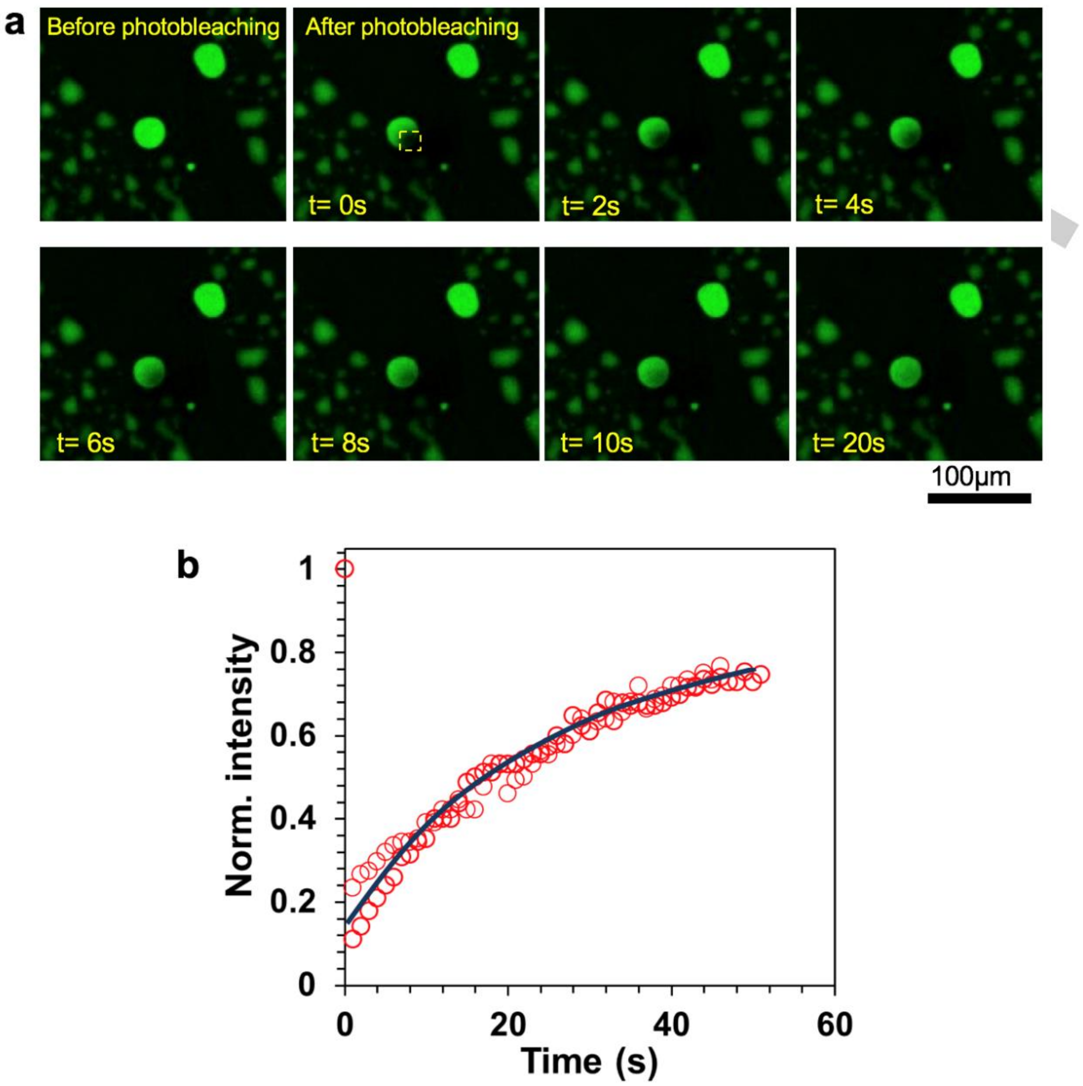

Figure S5. (a) Confocal images showing partial droplet bleaching and fluorescence recovery. (b) The FRAP recovery curves on basis of panel a. Coacervate droplets.compose 10 mM ATP and 10 mM pLys as well as 0.2 mg mL^{-1} FITC-pLys.

WILEY

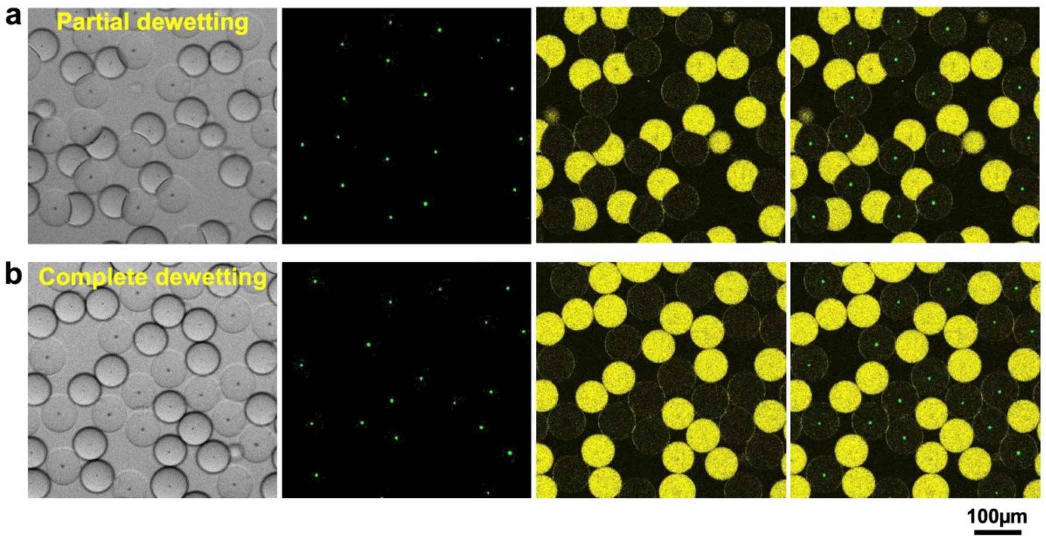

Figure S6. Confocal images of partially dewetted (a) and completely dewetted (b) double emulsion drplets with coacervate droplets inside. Images from left to right respectively are transmission channel, green fluorescent channel (labelled coacervated), yellow fluorescent channel (labelled liposomes and residual oil droplets) and overlay of green and yellow fluorescent channels.

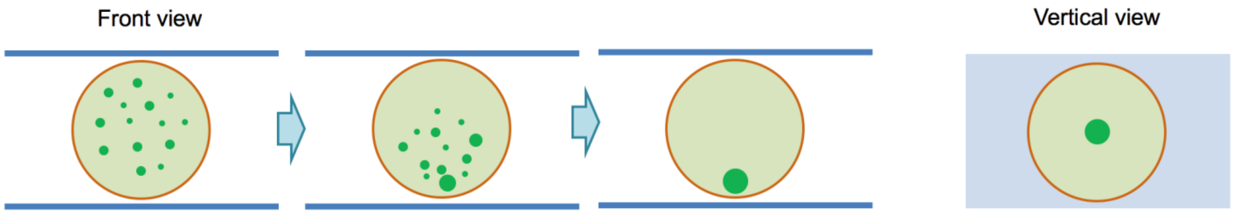

Figure S7. Schematics of fusion process of small coacervates into a big coacervate due to higher density of coacervate phase than that of the surounding solution.

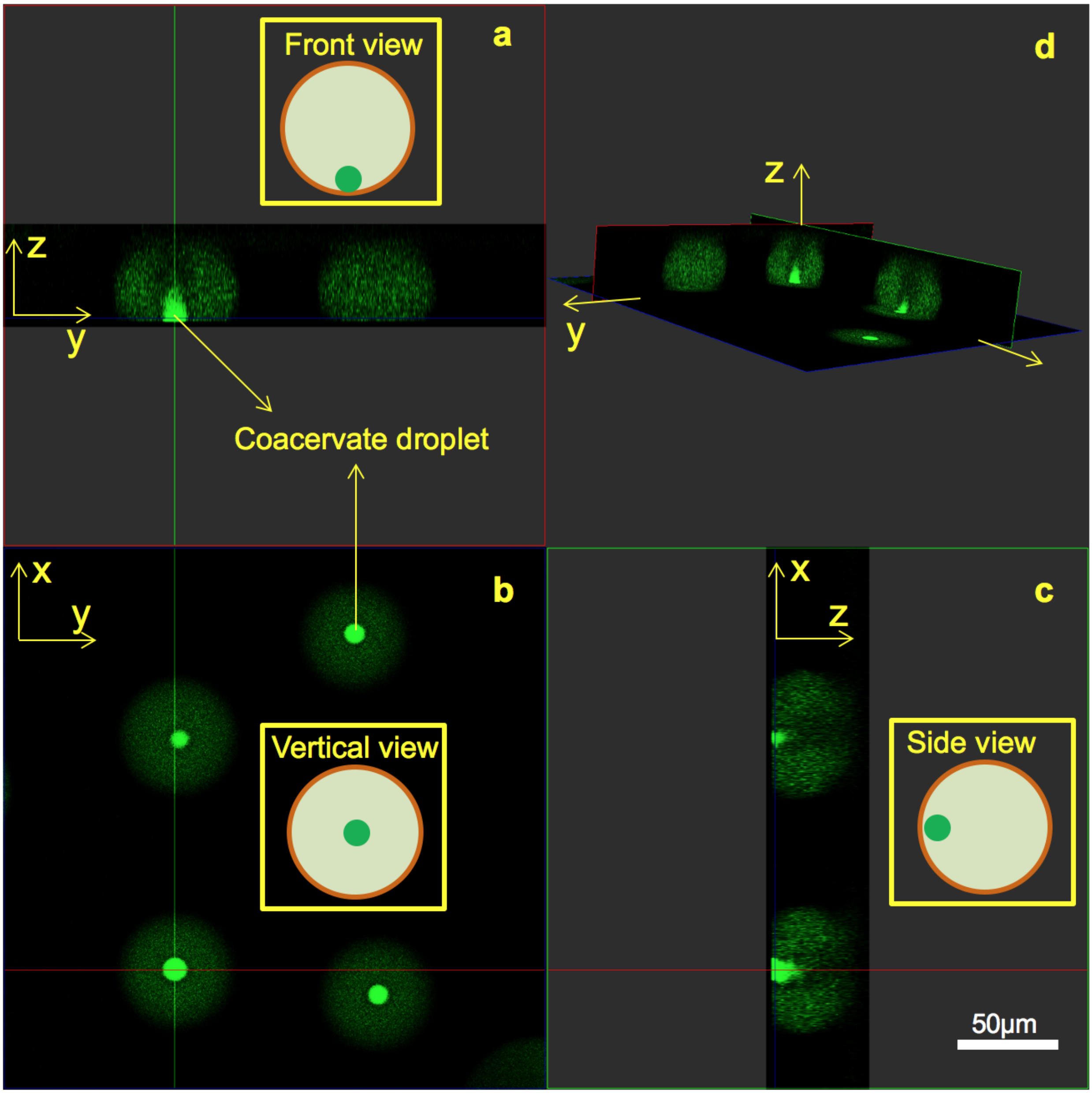

Figure S8. Front view (a), vertical view (b) and side view (c) of confocal images of liposomes containing single coacervate. (d) Reconstructed image showing the relevant slices in panels a, b and c, which were generated from a series of vertical slices.

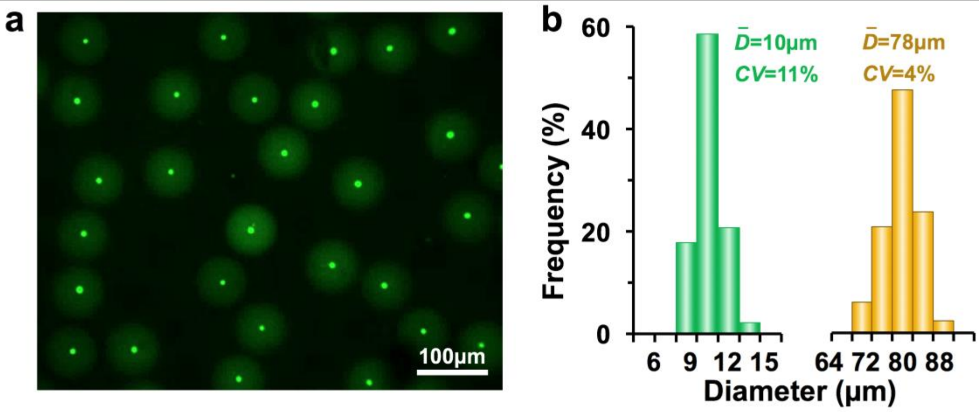

Figure S9. (a) Confocal images of labelled coacervate droplets inside lipsomes. (b) The size distribution of inner coacervate droplets and outer liposomes in panel a. Scale bar, 100 µm.

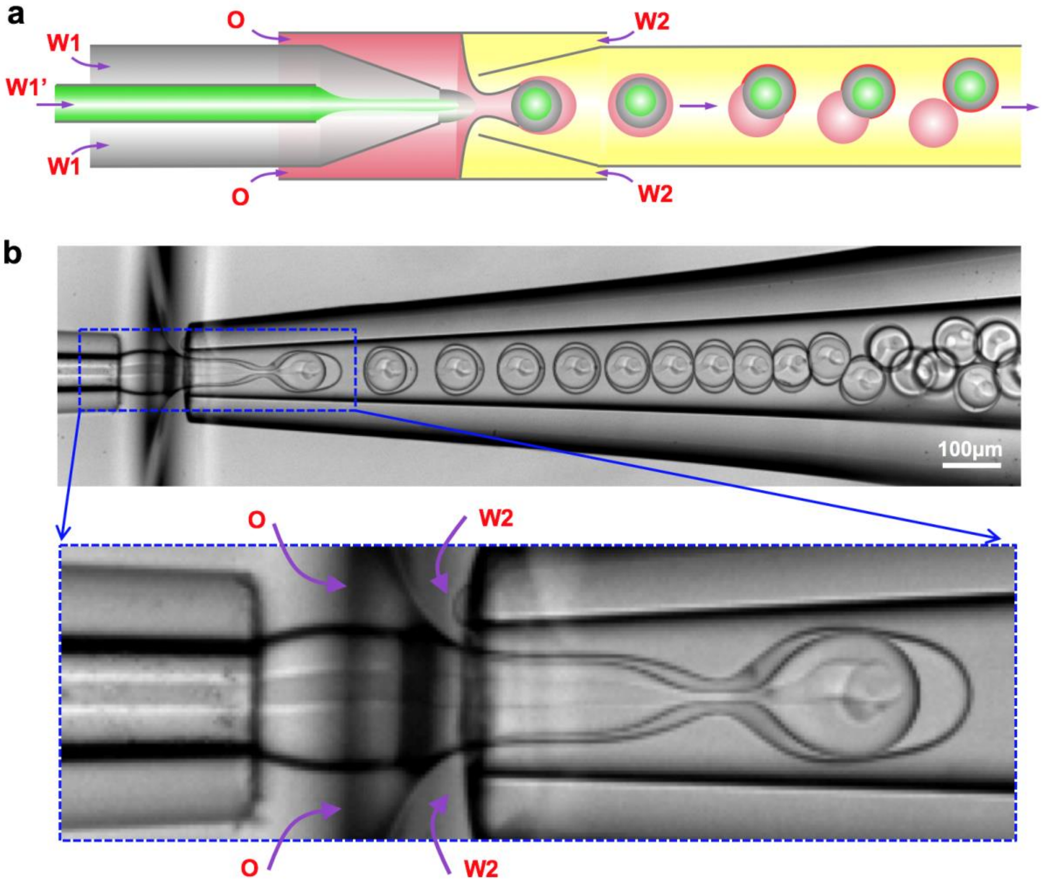

Figure S10. (a) Schematic illustration and (b) snapshots of encapsulation of W/W laminar flow jetting into double emulsion droplets to prepare templates for liposomes. Scale bar, 100 µm.

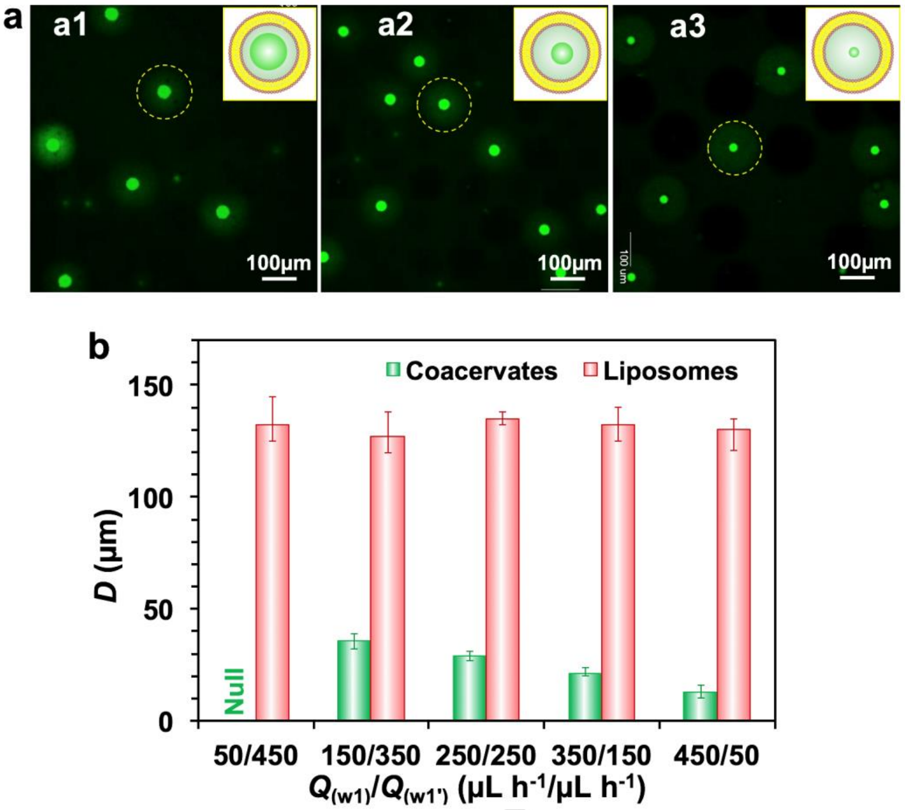

Figure S11. (a) Confocal images of lipsomes containing labelled coacervate droplets in different sizes, which were prepared by adjusting applied flow rates of phases W1 and W1'. (b) Influences of relative flow rates of two oppositely charged polyelectrolytes on the sizes of resultant coacervate droplets and liposomes, as $Q_{(O)}$ and $Q_{(W2)}$ were fixed at 500 µL h^{-1} and 5000 µL h^{-1}. Scale bars, 100 µm.

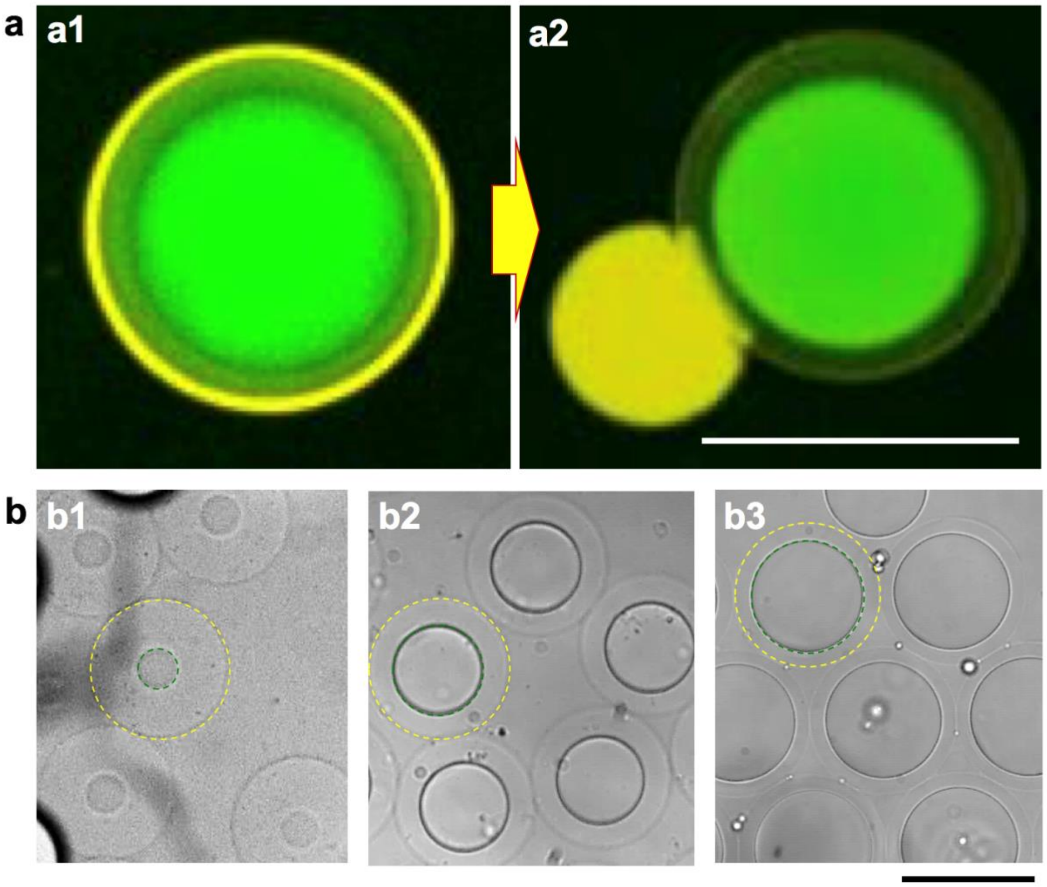

Figure S12. (a) Confocal images of a double emulsion drop with labelled PEG/DEX ATPS droplet inside an as-formed liposome. (b) Optical images of liposomes containing different-sized PEG/DEX ATPS droplets. Scale bars, 100 µm.

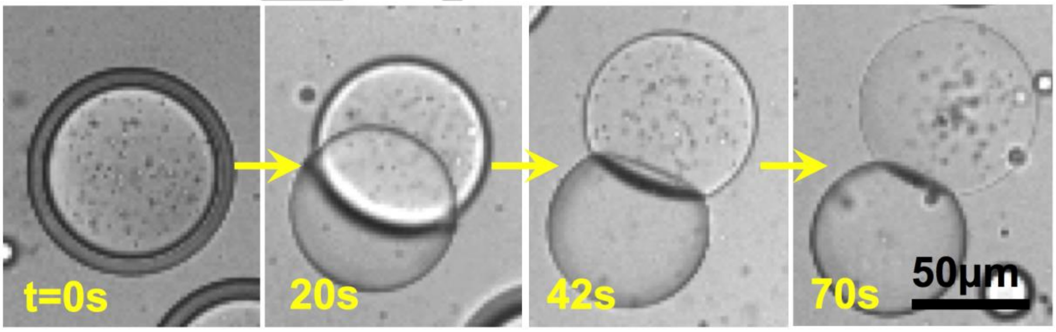

Figure S13. Optical image sequences show a quick deweting process of double emulsion droplets containing smaller coacervates inside. F-68 concentration in outer water phase is 1.0 wt%. Scale bar, 50µm

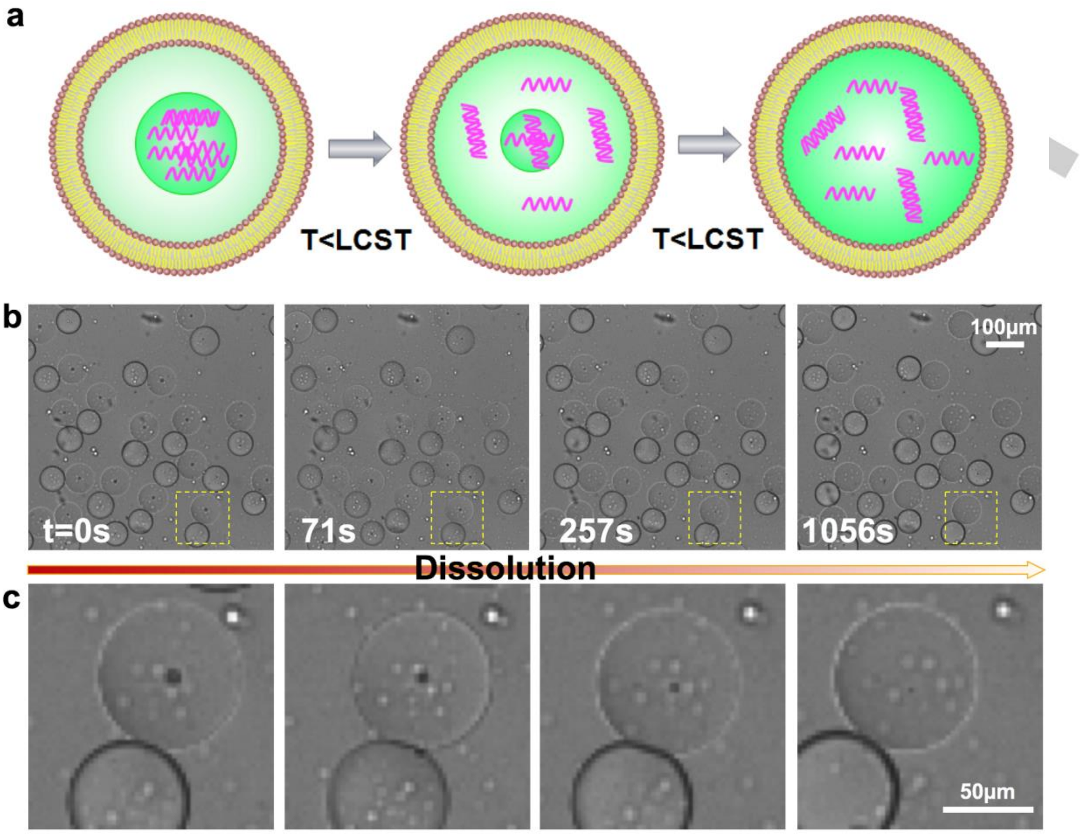

Figure S14. (a) Schematic illustration and (b,c) snapshots of dissolution process of coacervates in liposomes as temperature decreased lower than LCST (LCST=20 ^{o}C). T=16±1°C. Images in panel c are magnified views of dashed frames in panel b. Polycation: spermine, polyanion: polyU RNA. Scale bars, 100 μm

WILEY

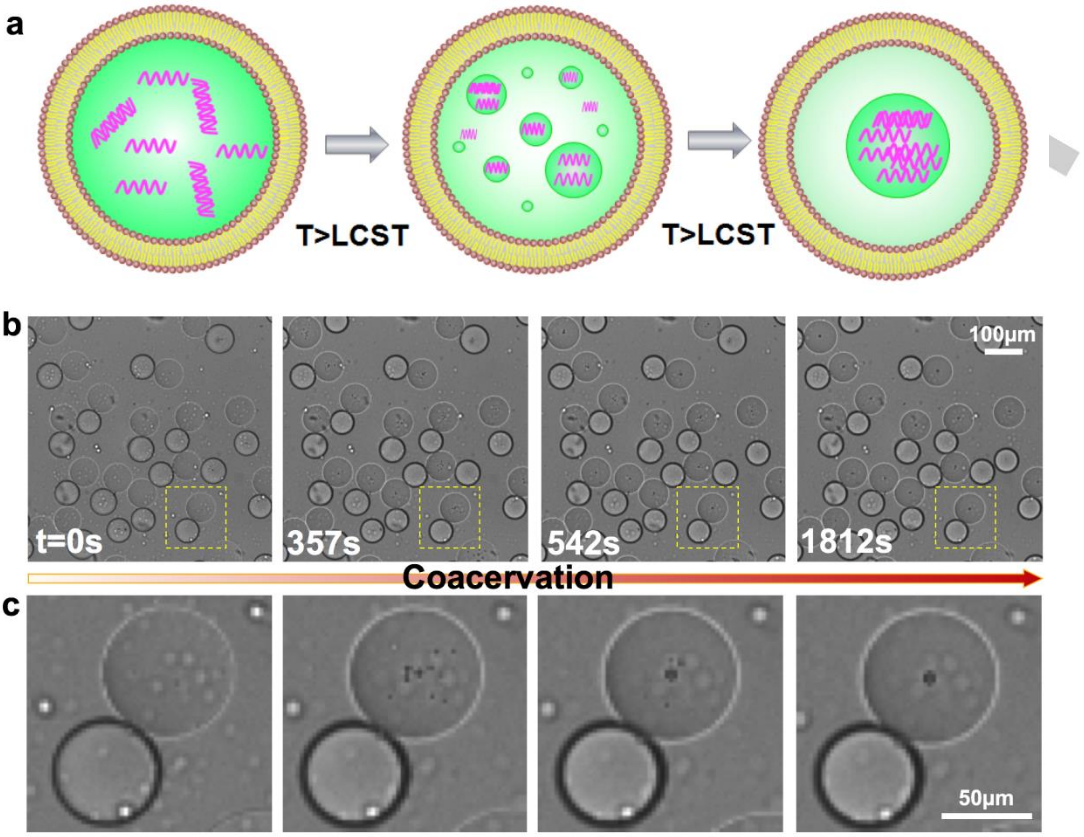

Figure S15. (a) Schematic illustration and (b,c) snapshots of coacervation process of coacervates in liposomes as temperature increased higher than LCST (LCST=20 ^{o}C). T=25±1°C. Images in panel c are magnified views of dashed frames in panel b. Polycation: spermine, polyanion: polyU RNA. Scale bars, 100 μm

WILEY

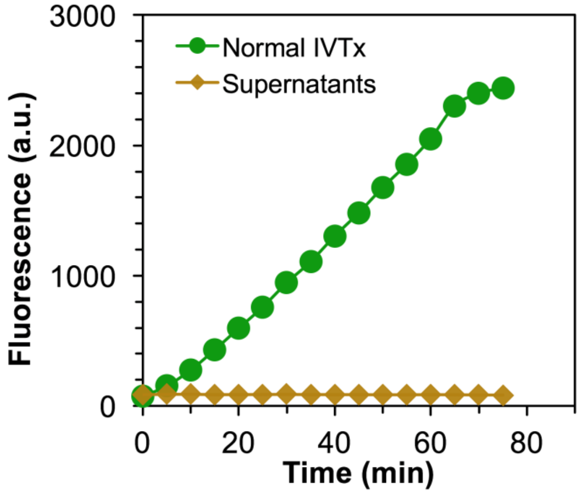

Figure S16. The expression kinetics of the normal IVTx and that in supernatants (coacervate removed) in bulk recorded by a plate reader.

Table S1. Coacervate systems were tested and successfully encapsulated in liposomes in our method.

No.	Polycation	Polyanion	Encapsulation in liposome (YES/NO)
1	Poly-L-lysine	ATP	YES
2	Poly-L-lysine	CoA	YES
3	Poly-L-lysine	Torula yeast RNA	YES
4	Spermine	Poly-U RNA	YES
5	PDDA	ATP	YES
6	Poly-L-arginine	CoA	YES

Part III. Supplementary Movies S1-S3

Movie S1. Microfluidic preparation of W/O/W double emulsion droplets loaded with coacervate systems.

Movie S2. Dewetting process & coalescence process to form single coacervate droplet in liposomes.

Movie S3. Temperature-induced reversible compartmentalization in liposomes.

WILEY-VCH

Part IV. Supplementary References

[1] C. Ma, Z. Tang, K. Wang, X. Yang, W. Tan, *Analyst* **2013**, *138*, 3013-3017.

[2] M. M. Hansen, M. Ventosa Rosquelles, M. Yelleswarapu, R. J. Maas, A. J. van Vugt-Jonker, H. A. Heus, W. T. Huck, *ACS Synth Biol* **2016**, *5*, 1433-1440.

WILEY-VCH
